# Enhanced sequestration of carbon dioxide into calcium carbonate using pressure and a carbonic anhydrase from alkaliphilic *Coleofasciculus chthonoplastes*

**DOI:** 10.1002/elsc.202100033

**Published:** 2021-07-26

**Authors:** Jonas Heuer, Yasemin Kraus, Marijan Vučak, An‐Ping Zeng

**Affiliations:** ^1^ Institute of Bioprocess and Biosystems Engineering Hamburg University of Technology Hamburg Germany; ^2^ SCHAEFER KALK GmbH & Co. KG Diez Germany

**Keywords:** calcium carbonate, carbon dioxide, carbonic anhydrase, high pressure, sequestration

## Abstract

CO_2_ in the atmosphere is a major contributor to global warming but at the same time it has the potential to be a carbon source for advanced biomanufacturing. To utilize CO_2_, carbonic anhydrase has been identified as a key enzyme. Furthermore, attempts have been made to accelerate the sequestration via pressure. This study aims to combine both approaches to achieve high sequestration rates. The carbonic anhydrase of the alkaliphilic cyanobacterium *Coleofasciculus chthonoplastes* (cahB1) and bovine carbonic anhydrase (BCA) are introduced into a high‐pressure reactor to catalyze the hydration of CO_2_ at up to 20 bar. The reactor is filled with a CaCl_2_ solution. Due to the presence of Ca^2+^, the hydrated CO_2_ precipitates as CaCO_3_. The impact of the carbonic anhydrase is clearly visible at all pressures tested. At ambient pressure a CO_2_ sequestration rate of 243.68 kg_CaCO3_/m^3^ h for cahB1 was achieved compared to 150.41 kg_CaCO3_/m^3^ h without enzymes. At 20 bar the rates were 2682.88 and 2267.88 kg_CaCO3_/m^3^ h, respectively. The study shows the benefit of a combined CO_2_ sequestration process. To examinate the influence of the enzymes on the product formation, the precipitated CaCO_3_ was analyzed regarding the crystalline phase and morphology. An interchange of the crystalline phase from vaterite to calcite was observed and discussed.

**Abbreviations**
ACC: amorphous calcium carbonate
BCA: bovine carbonic anhydrase
CA: carbonic anhydrase
PCC: precipitated calcium carbonate
SEM: scanning electron microscope
WAU: Wilbur‐Anderson units
XRD: X‐ray powder diffraction

## 1 INTRODUCTION

Climate change is identified as a major challenge of contemporary society. Driven by greenhouse gases in general, carbon dioxide (CO_2_) as the most common one is a principal contributor to global warming. Since the beginning of industrialization, human activities are responsible for an ongoing increase of CO_2_ in the atmosphere [1]. Considering the rapid growth of world's population and a continuous industrialization process in emerging industrial countries, the planet is strongly threatened by climate change and subsequently by natural disasters, increased sea level and temperature [2, 3]. In 2020, atmospheric CO_2_ reached consistently over 410 ppm which represents a nearly 50% increase compared to its pre‐industrial level (https://www.esrl.noaa.gov/gmd/ccgg/trends/global.html, November 2020).

On the other hand, this reservoir is a cheap and abundant carbon source, which is needed for a sustainable industrial biotechnology [4]. Current research focuses on the role of CO_2_ as a substrate either in a fermentative whole cell approach [5] or in an enzymatic system [6]. Both attempts aim to produce fuels or platform chemicals as valuable products. Therefore, not only the capturing but an efficient CO_2_ sequestration as a bioavailable C1 compound is a key challenge. When it comes to biological CO_2_ sequestration, carbonic anhydrase (CA) is proven to be a central enzyme [7]. Promising studies were made with CA in an active membrane which is able to separate CO_2_ out of a gas stream with high selectivity [8] or with CA as part of a multi‐enzyme microbead where CO_2_ capturing and subsequent processing take place in the same spot [9].

CAs are distributed over all forms of living organisms [10] and are essential for the transfer of CO_2_ and bicarbonate (HCO_3_^−^) by catalyzing the reversible hydration of CO_2_:

(1) $${\mathrm{CO}}_{2}+H_{2}O⇌\mathrm{HC}{O_{3}}^{-}+H^{+}$$

In detail, the water molecule attached to the zinc atom of the active center of the CA is deprotonated and initiates a nucleophilic attack. The target is the carbon atom of CO_2_ and the product is HCO_3_^−^ which is liberated in exchange to another water molecule in the last step [11]:

(2) $$Zn^{2+}-H_{2}O⇌H^{+}+Zn^{2+}-OH^{-}$$

(3) $$Zn^{2+}-OH^{-}+CO_{2}⇌Zn^{2+}-\text{HC}{O_{3}}^{-}$$

(4) $$Zn^{2+}-\text{HC}{O_{3}}^{-}+H_{2}O⇌Zn^{2+}-H_{2}O+\text{HC}{O_{3}}^{-}$$

An elegant way to sequestrate CO_2_ is to precipitate calcium carbonate (CaCO_3_) out of an aqueous solution containing Ca^2+^ ions. The solubility of CaCO_3_ in water is low and while reducing the amount of CO_2_, synthetic precipitated calcium carbonate (PCC) is a product used in various applications as filler material [12] pharmaceutical carrier [13], or nutritional supplement [14]. One reason for the broad application is its polymorphism. Within the different pure crystalline structures as calcite, vaterite and aragonite, many shapes as plates, rhombohedra, needles and spherulites are possible. In nature a great variety of organisms use biomineralization to build parts like shells and other structures [15, 16] Especially in corals and sponges calcium carbonate is a major material [17, 18]. For invertebrates and vertebrates CA plays important role in biomineralization. Besides being part of respiration and acid‐base balance processes [19], CA increases the calcification rate and manipulates the morphology. The whole mechanism is not completely understood but the potential motivates recent research [20, 21]. Organisms seem to control precipitation to calcite [22], vaterite [23] or aragonite [24] via CA.

**PRACTICAL APPLICATION**

The increasing amount of CO_2_ in the atmosphere is a major source for the climate change and global warming. At the same time, it is a huge reservoir of one‐carbon molecules. This potential has been addressed in the last years by setting up a roadmap to a biotechnological C1 economy. While the upcycling to valuable platform chemicals and fuels is making progress, an efficient CO_2_ sequestration remains a critical issue. In this study we investigated a combined approach. Carbonic anhydrase (CA) is used to sequestrate CO_2_ and catalyze the hydration into reactive HCO_3_^−^. The CA cahB1 from the alkaliphilic *Coleofasciculus chthonoplastes* was tested for this application. Additionally, the partial pressure of CO_2_ is increased to 20 bar to maximize the productivity. In this way, larger amounts of CO_2_ can be utilized once processes are scaled‐up.

For the precipitation, an alkaline pH is mandatory in consideration of the carbonic acid equilibrium system [25], because HCO_3_^−^ provided by CA can dissociate into carbonate ions (CO_3_^2−^):

(5) $${{\mathrm{HCO}}_{3}}^{-}⇌{\mathrm{CO}}_{3}{{}^{2}}^{-}+H^{+}$$

In the next step, if calcium ions (Ca^2+^) are present, they form CaCO_3_ together with CO_3_^2−^.

(6) $${\mathrm{CO}}_{3}{}^{2-}+{\mathrm{Ca}}^{2+}⇌\mathrm{CaC}O_{3}↓$$

Enzyme‐enhanced CO_2_ sequestration has been investigated in multiple manners: either by purifying and evaluating CAs from different origins [26, 27] or by immobilizing CA to make it more resistant to harsh conditions [11, 28]. Few studies dealt with the production of PCC under pressurized CO_2_. Montes‐Hernandez et al. [29] suggested an increased precipitation rate due to the increased solubility of CO_2_ while stating a limited impact of molecular CO_2_ on the precipitation itself.

To the best of our knowledge, these two approaches, the enzymatic and the pressure induced accelerations of CO_2_ sequestration process, have not been combined yet. Therefore, this study aims to evaluate the possible capture rate of CO_2_ by comparing the performance of two different CAs under different pressure levels. Because of the characteristics mentioned before, PCC is an interesting product itself. Consequently, the produced CaCO_3_ morphologies are characterized using SEM and XRD and the phase interchange during the precipitation process is investigated.

## 2 MATERIALS AND METHODS

### 2.1 Expression and purification of CA cahB1 from *Coleofasciculus chthonoplastes*

The gene *cahB1* was obtained from the group of Dr. Kupriyanova from the Russian Academy of Sciences (Moscow, Russia) who discovered the CA in the alkaliphilic cyanobacterium *C. chthonoplastes* (*ex‐Microcoleus chthonoplastes*) and fused it to thioredoxin [30] with a polyhistidin‐tag at the N‐ and C‐termini of the protein [31]. The amplified construct was cloned into the recombinant plasmid pET‐32b(+) (Novogen) and transformed into *E. coli* strain BL21(DE3). For CA production, the *E. coli* cells were cultivated at 37°C in LB‐medium until OD_600_ = 0.7 was reached and induced afterwards by adding 1 mM isopropyl thio‐b‐d‐galactoside (IPTG). The overproduction took place for 4 h at 25°C with a final OD of 3. The cells were separated from the fermentation culture broth by centrifugation and homogenized with a Spectronic SLM Aminco French pressure cell. The expressed proteins were purified by affinity chromatography using a Knauer FPLC equipped with a HisTrap HP column containing nickel ions resins purchased from Sigma‐Aldrich according to the manufacturer's protocol. Finally, the elution buffer was exchanged to purified water three times by recharging Amicon Ultra centrifugal filters with a molecular weight cut‐off of 30 kDa.

CA from bovine erythrocytes (BCA) (lyophilized powder, ≥2000 WAU/mg) was purchased from Sigma‐Aldrich.

### 2.2 Protein determination

The protein concentration was estimated performing the Bradford Coomassie brilliant blue assay [32] using bovine serum albumin as a standard and measuring absorbance at 595 nm.

### 2.3 Activity assay

The enzymatic activity assay of the alkaliphilic CA was performed according to Wilbur and Anderson [33]. In this assay CO_2_ is used as a substrate. Specifically, a CO_2_ saturated ice‐cold solution is prepared by bubbling pure CO_2_ into purified water. During the assay, the reaction vessel is tempered at 0°C. To start the reaction, 20 mL of ice‐cold CO_2_‐saturated water is given into 30 mL of a 20 mM Tris‐HCl mixture containing 15 μL purified water as a blank or 15 μL of enzyme solution. The Tris‐HCl buffer was adjusted to pH 8.3 at room temperature and then cooled down to 0°C. Wilbur‐Anderson units (WAU) are defined as the ratio between the time required to drop the pH from 8.3 to 6.3 for the enzymatic test subtracted from the blanks time T_0_ and divided again by the tests time:

(7) $$\frac{\mathrm{WAU}}{\mathrm{mL}}=\frac{T_{0}-T}{T*mL_{\mathrm{enzyme}}}$$

WAU is the standard unit for CA measured at 0‐4°C. All experiments were performed in triplicates.

### 2.4 Sequestration of CO_2_ into CaCO_3_ at ambient pressure

The ammonium carbonate diffusion method was adopted from Müller et al. [27] The carbonation of Ca^2+^ was performed at ambient pressure in a desiccator. In this method, CO_2_ is generated from ammonium bicarbonate (NH_4_HCO_3_) solution. The upper compartment of the desiccator contained beakers with 10 mL of 50 or 100 mM CaCl_2_ solution which was buffered to pH 9 with 25 mM Tris‐HCl. The experiments were executed either without enzymes or with 2 WAU/mL of BCA or cahB1. The desiccator was placed on a benchtop shaker with a frequency set to 50 rpm. Triplicates were run at room temperature for different time spans up to 5 h. The reactions were stopped at the indicated time by taking the beaker out of the desiccator and separate the solid phase from the liquid phase by centrifugation. Afterwards, precipitated CaCO_3_ is immediately washed with 2‐propanol and dried afterwards to prevent an on‐going phase interchange.

### 2.5 Sequestration of CO_2_ into CaCO_3_ at increased pressure

**FIGURE 1:**
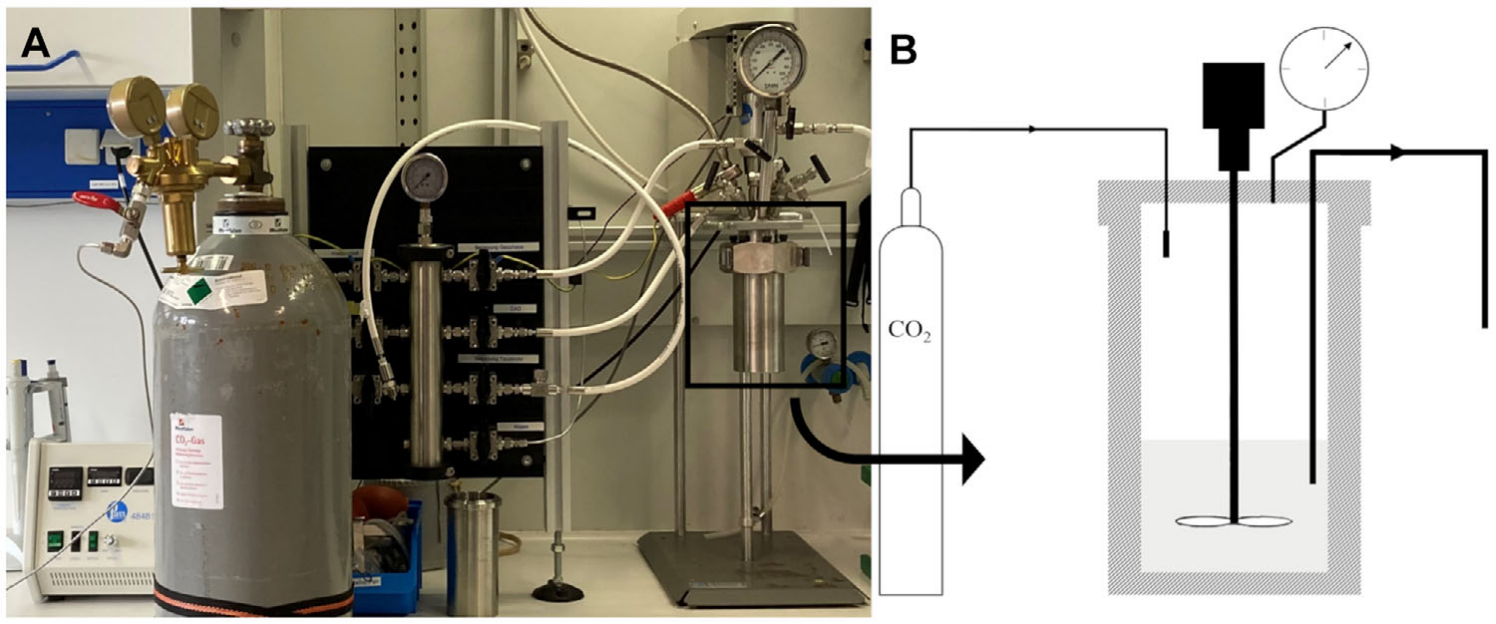
Parr‐reactor set‐up for the precipitation reactions under pressurized CO_2_ atmosphere. (A) is a photo of the whole set‐up and (B) the scheme of the reactor itself

Pressurized carbonation was performed in a high‐pressure reactor system of the Parr Instrument Company with an internal volume of 300 mL. A scheme of the set‐up is depicted in Figure 1. In parallel to the experiments conducted in the desiccator, 96 mL solutions of the same samples in terms of concentrations were prepared. The reactor was stirred at 50 rpm at room temperature. To start the precipitation, CO_2_ was injected into the system. CO_2_ was purchased from Westfalen Gas, Germany, with a purity of 99.99%.

The pressure was set to be constant at 5, 10, or 20 bar, meaning that CO_2_ adsorbed by the solution would be recharged to keep the pressure constant. The pressure is achieved by a gas cylinder with at least 50 bar CO_2_ inside and the system is flushed to remove all air to ensure a pure CO_2_ phase. Subsequently, the total amount of CO_2_ injected per run is the sum of CO_2_ in the gas phase, in the solution and precipitated as CaCO_3_ at the end of the run. The amount of CO_2_ in the gas phase can be calculated by the Peng‐Robinson equation of state [34], the solubility of CO_2_ is calculated later in Section 3.3 and the amount of CO_2_ in CaCO_3_ equals the initial concentration of Ca^2+^. For the runs with 50 mM CaCl_2_ this leads to 0.215 mol CO_2_ injected at 5 bar, 0.42 mol at 10 bar and 0.8 mol at 20 bar. Each run was repeated three times.

In order to determine the amount of precipitated CaCO_3_ and its characterization, samples of 1 mL were taken from the on‐going process through a valve. Depending on the pressure and the initial Ca^2+^ concentration the carbonations were run for up to 15 min until the reaction finished. The samples were treated as described before.

### 2.6 Determination of free Ca^2+^

To follow the precipitation of CaCO_3_ quantitatively, the concentration of the free Ca^2+^ in the supernatant is determined by ethylenediaminetetraacetic acid (EDTA) titration. In this complexometric titration, the endpoint is detected by a color change of the indicator Eriochrome Black T due to a lack of Ca^2+^ caused by the formation of Ca^2+^‐EDTA complexes [35]. Subsequently, the concentration of Ca^2+^ can be calculated using the volume of the supernatant $V_{Ca^{2+}}$, the concentration and volume of the EDTA solution $c_{\mathrm{EDTA}}$ and $V_{\mathrm{EDTA}}$, respectively:

(8) $$c_{\mathrm{EDTA}}\cdot V_{\mathrm{EDTA}}=c_{Ca^{2+}}\cdot V_{Ca^{2+}}$$

The conversion of free Ca^2+^ to CaCO_3_ can be calculated using the concentration of free Ca^2+^ in the beginning c_0_ and the determined concentration at a certain time c_t_.

(9) $$X=\frac{c_{0}-c_{t}}{c_{0}}\;=1-\frac{c_{t}}{c_{0}}$$

### 2.7 Characterization of formed CaCO_3_

The morphology of the dried CaCO_3_ was investigated using a scanning electron microscope (SEM) DSM 962 from Zeiss, Germany, operating at an accelerating voltage of 10 kV. The samples were suspended in ultrapure water, placed on a holder, dried again and sputtered with a layer of gold and palladium.

The qualitative phase analysis was performed by using X‐ray powder diffraction (XRD) measurements. A D8 Endeavor diffractometer from Bruker, Massachusetts, USA, was utilized to determine the crystalline structure using Cu Kα radiation (λ = 15,406 Å) and a 2 Theta angle ranging from 4° to 65°. The quantitative phase analysis was done by the Rietveld method [36] using Topas from Bruker AXS.

## 3 RESULTS AND DISCUSSION

### 3.1 Activity of cahB1

**FIGURE 2:**
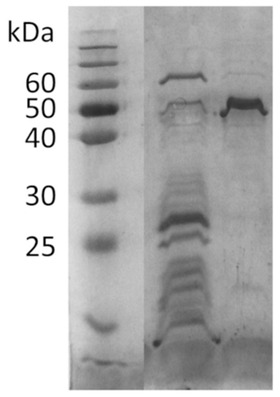
Twelve percent SDS‐PAGE showing overexpression and purification of cahB1. Lane A resolves the cell lysate of induced *E. coli* BL21(DE3) cells. The cahB1 protein complex is visible near the 50 kDa marker with a mass of approximately 50.3 kDa [31]. Lane B resolves cahB1 purified by affinity chromatography. Both lanes were loaded with a total protein mass of 10 mg

The successful production and purification of cahB1 was confirmed by electrophoretic analysis using SDS‐PAGE under denaturing conditions (Figure 2). The specific activity of the purified enzyme was determined to be 72.89 ± 3.28 WAU/mg in triplicates. In comparison, Kupriyanova et al. achieved 53.47 ± 4.88 WAU/mg [31]; however, using the total cell lysate. The issues regarding inclusion bodies reported by Kupriyanova et al. were not investigated in this study. The enzyme was stored in purified water at 4°C.

### 3.2 CaCO_3_ precipitation accelerated by carbonic anhydrase and pressure

**FIGURE 3:**
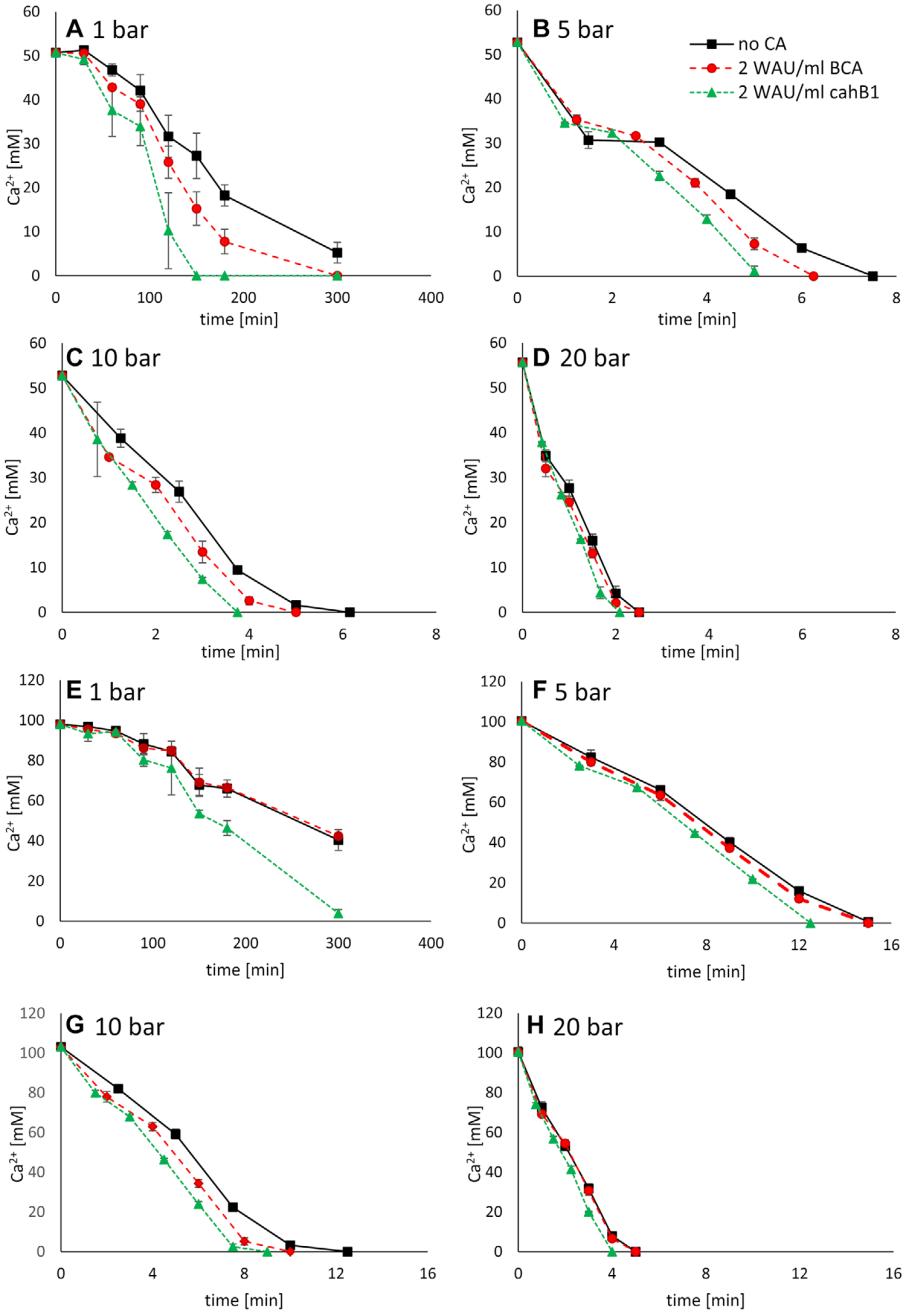
CaCO_3_ precipitation accelerated by CA. CaCO_3_ precipitated out of a 50 mM (A‐D) and a 100 mM (EH) CaCl_2_ solution in contact with a CO_2_ gas phase. For A‐D (with the exception of the x‐axis of A) the x‐ and y‐axis are bisected for the runs under pressure compared to E‐H to ensure proper differentiation between the enzymes. A and E refer to the runs at ambient pressure in a desiccator, B and F to the runs in the Parrreactor at 5 bar, C and G at 10 bar and D and H at 20 bar. The mean standard deviation is given

In general, the precipitation rate of PCC depends on the concentrations of the starting compounds Ca^2+^ and CO_2_ [37, 38]. To track the progress of the precipitation in different experiments, the removal of Ca^2+^ is plotted for the experiments with 50 mM (A‐D) and 100 mM (E‐H) CaCl_2_ solution in Figure 3. Please note the change in the scale of the x‐and y‐axis, for example it took roughly 2 min until no Ca^2+^ was left starting at 50 mM at 20 bar (3D) compared to 4 min at 100 mM and 20 bar (3H).

The time required (t_80_) for a removal of 80% of Ca^2+^ or 80% precipitation of CaCO_3_ was calculated using the exponential equation suggested by Stocks‐Fischer et al. [39] to describe microbial CaCO_3_ precipitation:

(10) $$c_{Ca^{2+}}=c_{0}\;-\frac{\Delta c_{i}}{1+e^{k*\left(t-z\right)}}$$

In the equation, Δc_i_ is the difference between the starting concentration c_0_ and the final concentration of $c_{Ca^{2+}}$, k the reaction rate, t the time and z the time point of the maximum of (dc/dt). The parameters k and z can be derived by fitting Equation 10 to the experimental results of Figure 3 and were determined using OriginLab (Version 2020) in this work.

$c_{Ca^{2+}}$ equals 0.2 since 80% of the Ca^2+^ are removed, c_0_ is 1 and Δc =_ _c_0_, because the reaction runs until all Ca^2+^ are removed, t_80_ can be calculated by rearranging Equation 10:

(11) $$t_{80}=\frac{\ln\left(\frac{1}{0.8}\right)}{k}+z$$

Table 1 shows the derived values of the parameters obtained from fitting the data of Figure 3 as described before. Generally, the greatest jump takes place between 1 and 5 bar where k increases by a factor of 10 to 30, while t_80_ is decreased by a factor of 20 to 40. Afterwards, the increased pressure seems to lead to a proportional or reverse proportional change of the parameters, respectively. Interestingly, at a concentration of 100 mM CaCl_2_ no catalytic effect is visible since the parameters are similar to the ones calculated without any enzyme. A possible explanation can be an inhibition caused by the Cl^−^ [40]. In the next section, t_80_ is used to calculate the sequestration rate of CO_2_.

**TABLE 1:** Parameters obtained from fitting the data of Figure 3 to the Stocks‐Fischer equation

$c_{Ca^{2+}}$ [MM]	Enzyme	P [bar]	K [min^‐1^]	Z [min]	T80 [min]
50	Without CA	1	0.023	150	159.7
		5	0.63	3	3.35
		10	1.14	2.5	2.70
		20	2.16	1	1.10
	BCA	1	0.032	120	126.97
		5	0.8	3	3.28
		10	1.173	2	2.19
		20	2.3	0.85	0.95
	cahB1	1	0.051	94.2	98.58
		5	0.88	2.5	2.75
		10	1.32	1.5	1.67
		20	2.7	0.85	0.93
100	Without CA	1	0.011	240	260.29
		5	0.4	7.75	8.31
		10	0.59	5.25	5.63
		20	1.15	2	2.19
	BCA	1	0.011	240	260.29
		5	0.4	7.25	7.81
		10	0.62	4.5	4.86
		20	1.15	2	2.19
	cahB1	1	0.02	162	173.16
		5	0.43	6.75	7.27
		10	0.68	4	4.33
		20	1.29	1.75	1.92

The different experimental conditions (concentration of Ca^2+^, type of enzyme and pressure) are listed to the corresponding reaction rate k, time point in the maximum of (dc/dt) z and the time until 80% of Ca^2+^ are removed t_80_. 2 WAU/mL of BCA and cahB1 were used, respectively.

### 3.3 Sequestration of CO_2_ into CaCO_3_

The improvement of the CO_2_ sequestration due to an enzymatic catalysis and an increased pressure was examined. Table 2 shows the production rate of CaCO_3_ and subsequently the rate of sequestered CO_2_. The results of this study refer to the experiments with an initial concentration of 50 mM CaCl_2_. For the calculation of the production rate, the starting concentration of CaCl_2_ plays a minor role. However, a broad range of anions and other small molecules seem to inhibit cahB1 including chloride [41].

**TABLE 2:** Production rates from the carbonations of the 50 mM CaCl_2_ solutions of this study compared to recent literature

Study	p [bar]	T [°C]	CA	Production rate [kg_CaCO3_/m_3_ h]
This study	1	RT	–	150.41
	5			745.98
	10			928.20
	20			2267.88
	1		BCA	189.18
	5			763.11
	10			1142.42
	20			2642.16
	1		cahB1	243.68
	5			908.70
	10			1499.16
	20			2682.88
Montes‐Hernandez et al. [29]	55	30	–	789.6
	90	90		213.17
Molva et al. [11]	1	RT	BCA	17.66
			BCA (immob.)	24.14
Müller et al. [27]	1	22	‐	33.36
			CA from *Sycon raphanus*	55.22 (3 WAU/mL)
				84.28 (10 WAU/mL)
Chafik et al. [26]	1	RT	CA from *Camelus dromedarius*	6.76

Different approaches in the experimental design make the results difficult to compare. Values from Molva et al., Müller et al. and Chafik et al. were not given in their publications but recalculated.

In fact, the different studies must be compared carefully. In this study and in the ones of Molva et al. [11] and Müller et al. [27], the solution is aerated across the surface while in the study of Montes‐Hernandez et al. [29] the gas is bubbled in and in the study of Chafik et al. [26] a solution is saturated with CO_2_ and afterwards mixed with a solution containing Ca^2+^.

As expected, the production rate of CaCO_3_ increases with the pressure according to Fick's law since the adsorption and hydration of CO_2_ were identified as the rate‐determining reaction step [25]:

(12) $$\psi \;=\frac{D_{L,\mathrm{CO}2}*\;a}{\delta }\;\left(C_{L,\;\mathrm{CO}2}^{*}-C_{L,\;\mathrm{CO}2}\right)$$

The diffusion flux ψ can be calculated using $C_{L,\;\mathrm{CO}2}^{*}$ the saturation concentration of CO_2_ in the liquid phase corresponding to its partial pressure ($p_{\mathrm{CO}2}$) in the gas phase, $C_{L,\;\mathrm{CO}2}$ the real CO_2_ concentration in the liquid phase, $D_{L,\mathrm{CO}2}$ the diffusion coefficient, δ the thickness of a “film” at the gas‐liquid interface, and the area of gas‐liquid interface. Assuming a pure CO_2_ gas phase, $C_{L,\;\mathrm{CO}2}^{*}$ can be calculated to be 33.3 mol CO_2_ per m^3^ of water at 1 bar and 22°C, 158.1 mol/m^3^ (5 bar), 306.7 mol/m^3^ (10 bar) and 574.3 mol/m^3^ (20 bar) using the software PHREEQC [42] at 50 mM CaCl_2_.

(13) $$C_{L,\;\mathrm{CO}2}^{*}=K_{H}\frac{\varphi_{CO_{2}}*\;P_{CO_{2}}}{\gamma_{CO_{2}}}$$

K_H_ is the Henry constant taken from the PHREEQC data, $\gamma_{CO_{2}}$is the activity coefficient in water and $\varphi_{CO_{2}}$the fugacity coefficient. Note that CaCl_2_ decreases the solubility of CO_2_ in water which is reflected in $\gamma_{CO_{2}}$. For 100 mM CaCl_2_, $\gamma_{CO_{2}}$ was found to be 1.07 [43]. Due to the model [44] applicable at concentrations below 3 M CaCl_2_, $\gamma_{CO_{2}}$ can be calculated to be 1.05 using linear regression at 50 mM CaCl_2_. More recently, the solubility was modelled for higher temperature, pressure and CaCl_2_ concentration [45] comparable to this study. The results show the expected impact at extreme conditions. The solubility increases approximately proportional within the partial pressure range of gaseous CO_2_ used in this study. At higher pressure, the fugacity coefficient starts to have a substantial influence on the solubility and slows down its growth. In PHREEQC, the Peng‐Robinson equation of state [34] is used to calculate the fugacity coefficient.

The production rate was calculated using the time t_80_ derived before, 80% of the initial concentration of Ca^2+^ and the reaction volume. The highest production rate was achieved at 20 bar when cahB1 was present (2682.88 kg_CaCO3_/m^3^ h). While the addition of BCA leads to a similar production rate of 2642.16 kg_CaCO3_/m^3^ h, in absence of an enzyme only 2267.88 kg_CaCO3_/m^3^ h were obtained. The presence of CA causes an increase of the production rate of 13.1% (BCA) and 20.6% (cahB1) compared to the chemical precipitation. At ambient pressure, the production rates are increased by 19.1% (BCA) and 60.1% (cahB1). At higher pressure, the increased solubility becomes the main driving force and at the same time the impact of the CA becomes less noticeable.

Montes‐Hernandez et al. achieved similar magnitudes by comparing a pressured gaseous CO_2_ to a supercritical CO_2_ precipitation out of a calcium hydroxide Ca(OH)_2_ solution as a Ca^2+^ source. The dissolution of Ca(OH)_2_ in water leads to the formation of two hydroxide ions resulting in an alkaline environment. The pH value above 12.5 in saturated solutions decreases the activity of all common enzymes or even causes denaturation. Compared to the pH optimum around 8 to 9 of BCA [45], cahB1 seems to be adapted to the alkaline environment with a pH optimum around 9 to 10 [31] but did not show significant activity in preliminary experiments with Ca(OH)_2_. On the other side, the pH of a CaCl_2_ solution can be easily adjusted using a buffer. Interestingly, the supercritical CO_2_ (90 bar, 90°C) led to a lower production rate of 213.17 kg_CaCO3_/m^3^ h than the gaseous CO_2_ (55 bar, 30°C) with 789.6 kg_CaCO3_/m^3^ h. According to the authors, the lower gas solubility caused by the higher temperature is the reason for the reduced production rate of the supercritical CO_2_ approach.

The approach Molva et al. is closer to the one executed in the desiccator. The Ca(OH)_2_ solution is in contact with a CO_2_ atmosphere through a defined gas‐liquid interface. As a central aspect, free BCA was compared with immobilized BCA. The production rates of the free and immobilized BCA were 17.66 and 24.14 kg_CaCO3_/m^3^ h, respectively. In this study, production rates of 150.41 (without CA), 189.18 (BCA) and 243.68 kg_CaCO3_/m^3^ h (cahB1) were obtained at 1 bar. Remarkably, the fluxes through the interface in the study of Molva et al. and in this study are similar (Molva et al.: free BCA 3.1 and immobilized BCA 4.1 mg_CO2_/cm^2^ min; this study: without CA 3.12, BCA 3.92 and cahB1 5.05 mg_CO2_/cm^2^ min). Therefore, a smaller interface to volume ratio probably caused the difference in the production rate.

Using the desiccator method, Müller et al. achieved 55.22 and 84.28 kg_CaCO3_/m^3^ h for 3 and 10 WAU/mL of a sponge CA, respectively. In this case, the lack of an agitation is most likely the reason for the lower production rates but the influence of the enzyme concentration is demonstrated. The increase of the CO_2_ sequestration rate due to the presence of CA is 66% for 3 WAU/mL in the study of Müller et al. As mentioned before, in this study 19% for BCA and 60% for cahB1 were achieved. Note that only 2 WAU/mL were used, the results are again in a similar range. Chafik et al. investigated a camel liver CA, resulting in 6.76 kg_CaCO3_/m^3^ h. The experimental setup deviates but a high sequestration capacity was achieved compared to other works studying mammalian CA. Therefore, the results are included to give an overview.

### 3.4 Characterization of formed CaCO_3_

**FIGURE 4:**
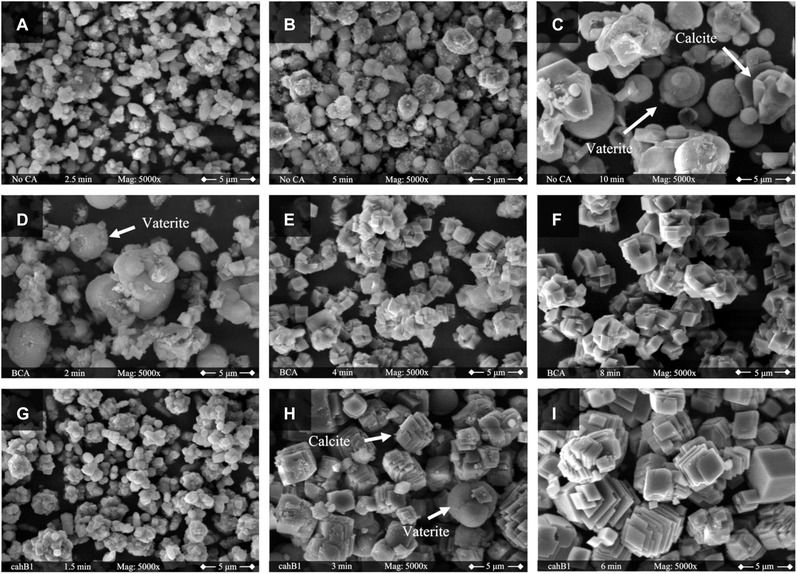
SEM images of the crystals formed during the experiments at 10 bar with a CaCl_2_ concentration of 100 mM. Pictures on the top panel correspond the experience without CA. The sample in A is taken 2.5 min after the beginning of the reaction and shows agglomerates of small particles. B (5 min. after beginning of the reaction) shows agglomerates of spheres mainly, with increased in size compared to A and can be identified as vaterite. After 10 min, a mix of spheric and rhombohedral crystals with a diameter around 5 μ can be found in C. In the middle panel, the results with 2 WAU/mL BCA are shown. The solid phase in D appears more mature compared to A since the particle size is increased and spherical vaterite is present. Smaller particles appear to have already a typical rhombohedral calcite shape. In E and F agglomerates of calcite crystals are dominant which increase in size during the time of the experiment. The SEM images on the bottom panel are taken from samples of the experiment with 2 WAU/mL cahB1. G is comparable to the small agglomerates of image A while H shows calcite particles with some remaining spherical vaterite. After 6 min, I shows again the final calcite particles distinctively stepped at the edges

To investigate the formation of CaCO_3_, the solid phases were characterized by using SEM and XRD. Due to similarity of CaCO_3_ particles from 50 mM to CaCO_3_ particles from 100 mM CaCl_2_ solution regardless of a pressure of 5, 10 or 20 bar, not all samples are analyzed and discussed. The SEM images of the trials at 10 bar and 100 mM CaCl_2_ are shown in Figure 4. Studies dealing with CA involved in the CO_2_ sequestration and CaCO_3_ precipitation focus on the morphology of the final product after the whole precipitation process is complete. In this work, the interchange of crystalline characteristics by taking and analyzing samples at different steps of the precipitation process was examined.

**FIGURE 5:**
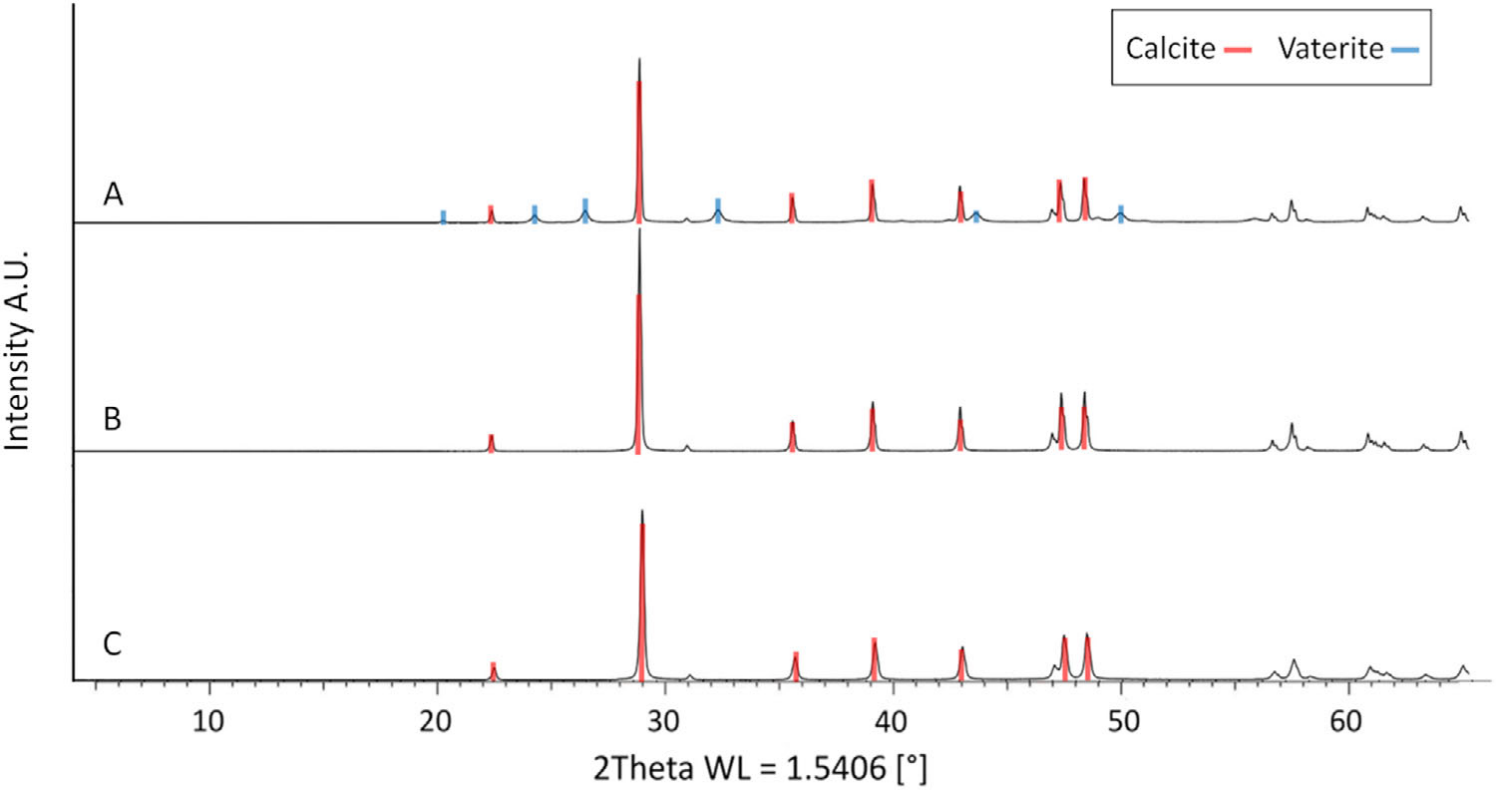
XRD patterns of the precipitated CaCO_3_ particles from the experiments at 10 bar and 100 mM CaCl_2_ in absence of CA (A), with BCA (B) or cahB1 (C). Calcite phase diffraction peaks are marked in red and vaterite phase diffraction peaks are marked in blue

In the absence of CA, undefined slightly agglomerated particles with a size below 1 μm are present after 2.5 min of reaction time (Figure 4A). Figure 4B shows grown particles after 5 min which start to look like spherical vaterite covered with a “dusty” layer. After 10 min (Figure 4C) spherical vaterite particles are grown to up to 5 μm. In the classical approach nucleation takes place in a supersaturated ionic solution where meta‐stable clusters are formed and decomposed, making the creation of a crystalline precursor which overcomes the critical cluster size a stochastic event. Modern theories favor a pathway of stable prenucleation clusters of ions, which can appear in undersaturated solutions as well, leading to an amorphous phase [46]. In the further course, the amorphous phase can translate to one of the water‐free phases aragonite [47], vaterite or calcite [48]. Therefore, the observable structures in the beginning may be the result of an on‐going interchange of amorphous calcium carbonate (ACC) to vaterite. A study in a similar system promotes a phase change from ACC to vaterite starting within the first minutes depending on the temperature [48]. The increased reaction speed due to the pressure supports the formation of ACC but at the same time a pure ACC phase is unlikely to observe. At the end (Figure 4C), the interchange to calcite rhombohedra is taking place [49]. The finale state can be proven by the XRD pattern of the sample (Figure 5A) [50]. Calcite phase diffraction peaks are present at 2θ = 23°, 29°, 36°, 39°, 43°, 47°, 49° and 57° with its characteristic peak at 2θ = 29°. Meanwhile, vaterite phase diffraction peaks appeared at 2θ = 21°, 25°, 27°, 33°, 44° and 50°. The quantitative phase analysis using the Rietveld method leads to a 60% fraction of calcite and a 40% fraction of vaterite.

In the presence of 2 WAU/mL BCA (Figure 4D), spherical vaterite with a diameter of 5 μm appears earlier at 2 min. According to the results described before, the precipitation reaction seems to be accelerated as well as the crystalline phase transformation. Again, the smaller particle agglomerated might be a late stage of ACC, in this case the cubic shape could refer to calcite. After 4 min (Figure 4E) the conversion to calcite is already finished and until 8 min (Figure 4F) the agglomerates grow to a size of around 5 μm. In the corresponding XRD pattern (Figure 5B) mere calcite phase diffraction peaks appear as expected.

The third panel shows PCC performed in the presence of 2 WAU/mL cahB1. In the beginning (Figure 4G), a solid phase like the one in the beginning of the one in absence of CA is present. As mentioned before, it might be an amorphous precursor of calcite and/or vaterite. In Figure 4H rhombohedral calcite is the dominant phase with only a few spheric vaterite particles left. Again, this observation supports the assumption of a fast, morphologic interchange due to the present of carbonic anhydrase. After 6 min, a pure calcite phase is visible (Figure 4I) proofed by the pattern of the XRD measurement (Figure 5C). In this SEM image the edges of the cubic‐like calcite particles appear more stepped compared to the shapes discussed before. Functional groups of proteins are expected to inhibit the growth of calcite at increased concentrations [51] and similar effects of shrunk edges can be found in many studies focusing on the biomineralization [52, 53, 54]. However, since the enzyme concentration is low in this study, the effect plays a minor role.

**FIGURE 6:**
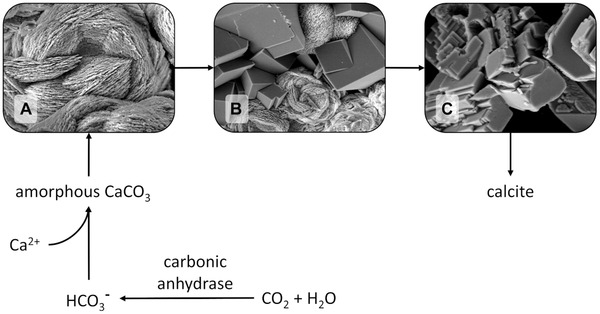
Flowchart of the observed precipitation process of calcite via an intermediate vaterite phase. pCO_2_ as a driving force shifts the equilibrium reaction strongly to the product side. Panel A shows a pure vaterite phase, B a vaterite‐calcite mix phase and C the final pure calcite phase

Former studies suggested a change from vaterite to calcite by a dissolution‐reprecipitation aging mechanism [30, 33]. The first generation of particles is (partly) dissolved and reprecipitated again to new more stable morphology. In the present composition, the higher solubility of vaterite compared to calcite [48] is the driving force in the process at ambient conditions. Therefore, in the final phase of the precipitation the solution is supersaturated regarding calcite but not vaterite. Subsequently, the phase shifts from vaterite to calcite with time. The mechanism supported by this study is highlighted in Figure 6. The high pressure and the catalysis of CO_2_ to HCO_3_^−^ by CA leads to a highly supersaturated solution and a fast precipitation rate. As mentioned before, ACC is expected to be part of the process. The packing density of Ca^2+^ in AAC is similar to the one in crystalline forms like vaterite and calcite, making an interchange to this phases possible [55]. In the three SEM images the dissolution‐reprecipitation aging is illustrated. The surface of the vaterite is dissolved and reprecipitates as calcite. This process continues in aqueous solution, until a pure stable calcite phase remains.

In contrast to the SEM images from samples taken from the Parr‐reactor, the particles obtained from the precipitation in the desiccator did not show a systematic pattern.

**FIGURE 7:**
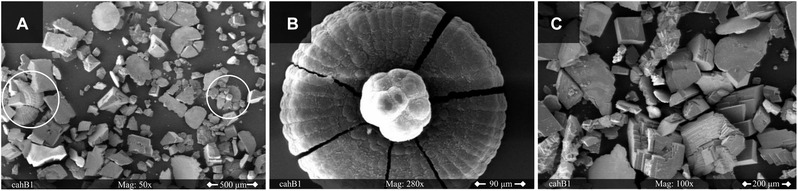
SEM images of the crystals formed during the experiments in the desiccator at ambient pressure with a CaCl_2_ concentration of 100 mM and in the presence of cahB1. The white circles in A indicate fragments of flower‐like morphologies as enlarged in image B. In C calcite agglomerates with one flat surface are present

The solid phases in Figure 7 look like typical calcite rhombohedra which are magnitudes larger than the particles found in the Parr‐reactor. A cause might be the longer reaction time, resulting in the conversion into calcite before the first sample was taken. Furthermore, the aging and growing of the particles is more present because the slight supersaturation leads to stable crystals. On the other side, the high supersaturation of the pressure experiments leads to the rapid nucleation of multiple precursors. It is interesting to note that many agglomerates in Figure 7C show a smooth flat surface on one side, indicating that they were located at the glass wall of the beaker or at the gas‐liquid interface. This was not visible in the Parr‐reactor samples, consequently the fluid mechanics and therefore the mass transport should be compared carefully between both approaches.

In addition, some flower‐like crystallographic structures (Figure 7B) appeared in the desiccator experiments. This unusual phenomenon does not seem to be an artefact since fragments of similar flowers can be found in 6A highlighted by the white circles. In fact, these agglomerates were described before [56]. They were found when precipitating CaCO_3_ on the inner surface of an eggshell membrane. The composition of a vaterite sphere in the middle surrounded by calcite petals was suggested by Takiguchi et al. and is supported by the SEM images obtained in this study. The structure might be the result of a covering process leading to capsulated vaterite particles.

## 4 CONCLUDING REMARKS

For the first time, the approach of using CA for biological CO_2_ sequestration was performed in a high pressure reactor with up to 20 bar. Additionally, a novel CA (CA cahB1) from the alkaliphilic cyanobacterium *C. chthonoplastes* was shown to be a promising enzyme showing a higher CO_2_ sequestration rate than the mostly used BCA. An explanation is the difference of the pH tolerance of both enzymes. The adaptation to the alkaline environment of cahB1 is advantageous even if the physiological reason is not discovered yet. Since the precipitation of CaCO_3_ highly depends on the presence of CO_3_^2−^ a more acidic pH would not increase the total reaction rate of the CO_2_ sequestration. The highest CO_2_ sequestration rate of 2682.88 kg_CaCO3_/m^3^ h for cahB1 was achieved at 20 bar. The rate is higher than the ones reported so far using CA or pressurized CO_2_ alone. Interestingly, the enhancement of CO_2_ sequestration of the CA decreased with increasing pressure, in case of cahB1 from 60.1% at ambient pressure to 20.6% at 20 bar. Subsequently, further increased pressure can lead to even higher sequestration rates but demands for higher enzyme concentrations. The next step is to develop suitable devices which can applicate the CO_2_ sequestration technology and link it to additional reactions for upcycling. The increased process costs associated with “single use” of purified enzymes may be addressed using approaches like enzymatic liquid membranes and other immobilization techniques. It is understood that cost efficiency is an economic challenge of enzyme‐aided CO_2_ sequestration.

Additionally, the precipitation of CaCO_3_ under pressure and influence of CA was investigated and an interchange of the crystalline phase from vaterite to calcite was observed in detail. As a precursor, amorphous calcium carbonate is expected to play a major role in highly supersaturated solutions but could not be identified without doubt. Future work should focus on the very beginning of the precipitation.

## CONFLICT OF INTEREST

The authors declare no conflict of interest.

## Data Availability

The data that support the findings of this study are available from the corresponding author upon reasonable request.
